# Changes in the Aromatic Compounds Content in the Muscat Wines as a Result of the Application of Ultrasound during Pre-Fermentative Maceration

**DOI:** 10.3390/foods10071462

**Published:** 2021-06-24

**Authors:** Fátima Aragón-García, Ana Ruíz-Rodríguez, Miguel Palma

**Affiliations:** Department of Analytical Chemistry, Center of Agri-Food and Wine Research (IVAGRO), Faculty of Science, University of Cadiz, 11510 Puerto Real, Spain; fatima.aragon@uca.es (F.A.-G.); miguel.palma@uca.es (M.P.)

**Keywords:** aroma, ultrasound, pre-fermentative maceration, wine, muscat

## Abstract

This research focuses on the aromatic composition of Muscat of Alexandria wines after the application of ultrasound for 40 or 80 min during a 4 h pre-fermentative maceration process. Two methods of ultrasound application were compared in this study: probe ultrasound and bath ultrasound, for periods of 10–20 min per hour. Increases of more than 200% were obtained for some of the compounds from the skins, such as two of its terpenes, citronellol and nerol. On the other hand, increases in alcohol and ester values were registered with the application of ultrasound for 40 min. However, a significant decrease in these compounds was recorded when the ultrasound process was extended. In fact, when ultrasound was applied for 80 min, content values were even lower than those registered for the wine produced without the application of ultrasound. At the sensory level, the effect resulting from probe and bath ultrasound application for different times were compared, where most of the judges successfully discriminated the wines resulting from the application of ultrasound bath. According to data, the wines resulting from the application of ultrasound bath for 80 min presented the most significant differences, which affected the aromas of white fruit, tropical fruit, stone fruit, flowers and citrus.

## 1. Introduction

The varietal aroma of wine is one of the most sought-after sensory characteristics when elaborating young wines. The aromatic characteristics of wine depend on the starting grape and on the winemaking process. The compounds responsible for aroma are found in both the pulp and the skins, even if they are found at higher levels in the skins [1,2]; therefore, a winemaking process that allows the extraction of these compounds, usually during the pre-fermentative stage, should favor the varietal character of the final wine [3].

The extraction of aromatic compounds is achieved by maceration of the grape skin with the must. For red wine, the duration of this maceration is similar to that of the fermentative period since it is designed primarily for the recovery of the anthocyanins responsible for its red color. During that time, the contact between the solid parts—particularly the skins—and the must favors the diffusion of the aromatic compounds from the skins to the must [4]. Even for red wines, some techniques are used to increase the extraction of different compounds from the grape skins [5,6,7,8]. However, when making white wines, long maceration processes are not very common since oxidative processes and the co-extraction of non-interesting or even undesirable compounds into the final wine may occur [9].

The cryomaceration or cold maceration technique increases, on the one hand, the concentration of aromatic compounds [10], although, at the same time, it favors the extraction of other components that could confer undesirable character to the wine [11]. A related technique is cryo-freezing or cryoextraction. By freezing the berries, the cells of the skins are broken and, when they return to room temperature, the compounds located in the solid parts are more easily released into the medium [12].

The application of high-intensity ultrasound during the pre-fermentative maceration process is another interesting alternative. The aim is to accelerate the diffusion rate of the compounds from the skins into the must. It is assumed that ultrasound contributes to cell autolysis (cell disintegration) and facilitates the release of the compounds into the medium [13,14,15]. Similarly, some studies have been carried out on the application of different frequencies during the production of red wines, where an increase in varietal compounds content was obtained, particularly during the fermentative phase, when the must remains in contact with the solid parts [16]. There are two most common ways to apply ultrasound: the first one consists of submerging a probe in the must container, while the second one involves submerging the container in a bath in which ultrasound is applied. This paper compares these two ultrasound application methods, and although both methods transmit ultrasound to the medium, it has been proven that there can be important differences regarding their outcome [17]. Along with the study of the application method, the effect of different application times during the pre-fermentative maceration for the winemaking from Muscat of Alexandria was evaluated.

Muscat of Alexandria is a variety characterized by a high percentage of combined and, above all, free aromatic components [18]. Terpenes, which are mostly distributed in the inner part of the skin, and to a lesser extent in the pulp, are the compounds responsible for the varietal aroma [2,19,20]. The main terpenes described in this variety are linalool, citronellol, nerol, geraniol and α-terpineol [15,21].

## 2. Materials and Methods

### 2.1. Varietal and Wine Making

To evaluate in a simple way the effects of ultrasound application, the Muscat of Alexandria variety, a grape variety with a high aromatic component, was used. The harvest date was established according to a grape acidity corresponding to 6.15 g L^−1^ of tartaric acid and a sugar level of 9 °Be.

After the grapes were received, they were stored for 12 h at 4 °C, after which time they were destemmed and crushed to obtain a mixture of must and paste. The mixture was placed in 50 L tanks where it underwent a cold pre-fermentative maceration for 4 h at 4 °C. This period is much shorter than the usual 3–5 day period for cold maceration [10]; however, it was expected that ultrasound would increase the extraction rate.

After the maceration, the must was pressed and a static settling for 24 h at a temperature of 4 °C was applied. This was followed by targeted alcoholic fermentation by adding 25 g hL^−1^ *Saccharomyces cerevisiae* var. bayanus yeasts, commercially knows as “viniferm PDM” (Agrovin, Ciudad Real, Spain), which was used for the fermentation process in 50 L containers. The fermentation started 24 h after the inoculation of the yeast and was carried out for 7 days, never exceeding 14 °C. Lastly, the wines were statically racked for 5 days and filtered through 20 µm pore size cellulose filters.

### 2.2. Ultrasound Application

The ultrasound technique was applied in different periods over the four hours of the pre-fermentation maceration process. Previous experiences during winemaking process for both white and red wines have reported that about 2 h of ultrasound application during the maceration step provides significant effects on the final wines [22,23,24]. Two variables were studied in this case: ultrasound application method and application time.

Ultrasound application methods:

1. *Ultrasonic bath* (USB) (Model ACM-200E, Ultratecno, Massalfassar, Spain). The tanks were submerged in a bath with water at 4 °C and ultrasound was applied to the system.
2. *Ultrasonic probe* (USP) (Model UIP 1000hdT, Dr. Hielscher, GmbH, Berlin, Germany). The tanks were sonicated by submerging the ultrasonic probe directly into the tank containing the must, with external temperature control at 4 °C.

Ultrasound application times:

1. 10 min h^−1^ tests (USB 40, USP 40). Over the 4 h of pre-fermentative maceration, the tanks were subjected to ultrasound for 10 min periods every hour, up to a total of 40 min.
2. 20 min h^−1^ tests (USB 80, USP 80). Over the 4 h of pre-fermentative maceration, the tanks were applied ultrasound for periods of 20 min every hour up to a total time of 80 min.

All the tests were carried out in duplicate, and in parallel, a control wine (WU) was elaborated using a 4 h pre-fermentative maceration, also in duplicate, under the same temperature conditions as the other tests but without the application of ultrasound.

After the application of ultrasound, all wines showed very similar values of pH, ranging from 3.42 for USP80 to 3.45 for USB80 with non-significant differences (*p* < 0.05). The final ethanol levels were also similar for all wines, ranging from 10.6 for WU to 10.7 for USB40, with non-significant differences (*p* < 0.05). Volatile acidity was also determined for all wines; it ranged from 0.29 g L^−1^ for WU to 0.32 g L^−1^ of acetic acid, however with non-significant differences (*p* < 0.05).

### 2.3. Characterization of the Wine Samples

The effects of ultrasound in the volatile composition were evaluated using free terpene derivatives, some hydroxylated compounds and some ethyl esters because they provide direct information about the effects of ultrasound during the maceration. Glycosidated compounds were not considered to avoid any additional sample treatment that could increase the variability in the analyses.

The aromatic compounds found in the final wines produced with or without ultrasound were studied using Solid Phase Extraction and Gas Chromatography-Mass Spectrometry (SPE-GC-MS), following the method described by Piñeiro [19]. A Visiprep SPE vacuum manifold with 12 ports by Supelco was used to perform up to 12 extractions simultaneously. For the extraction, prior to use, 3 mL cartridges (Strata SDB-L, Phenomenex, Torrance, CA, USA) were conditioned by rinsing them with 4 mL of dichloromethane, 4 mL of methanol and finally with 4 mL of an ethanol–water mixture (12%, v/v). Then, 50 mL of wine was flushed through the cartridge by vacuum suction (−0.67 atm). The cartridge was cleaned up by flushing 10 mL of water. The cartridge was then dried under vacuum (−0.67 atm). Finally, the compounds were eluted using dichloromethane (2 mL). The separation and quantification of the volatile compounds were performed on a GCMS-TQ8040 (Shimadzu Corporation, Kyoto, Japan) equipped with a 60 m × 0.32 mm i.d. fused silica capillary column coated with SupraWax-280 (Teknokroma, Barcelona, Spain). The separation conditions were as follows: injector temperature 200 °C; GC column temperature 40 °C (5 min) at 2 °C min^−1^ up to a final temperature of 230 °C (20 min); the carrier gas used was He at 40 kPa.

A recovery study was performed, and those compounds with recovery percentages greater than 85% by the SPE-GC-MS method were analyzed [19]. Three samples were used: (a) an ethanol:water 10% matrix with an addition of 200 µL of standard sample, (b) a wine sample with 200 µL of the standard sample added, (c) a pure wine sample. This recovery study was performed in triplicate, and results can be found in the Supplementary Materials (Table S1).

The peaks obtained in the chromatogram were identified using the NIST14 library (National Institute of Standards and Technology, Gaithersburg, MD, USA). The peaks were quantified by means of a calibration line based on standards of known concentration that were prepared in a hydro-alcoholic matrix (10% ethanol in water adjusted to pH = 3.5 using tartaric acid). Table 1 shows the data for the calibration curves. The quantification limits are similar to those found in the literature [25,26]. Each component was quantified at the most intense m/z of its mass spectrum.

### 2.4. Odor Activity Value (OAV) and Sensory Analysis

The Odor Activity Value (OAV) is the ratio between the concentration of a compound and its detection threshold. When OAV is greater than 1, it indicates that the compound individually influences the aroma component; however, when it is less than 1, it implies that, individually, this aroma will not be perceived, but it may influence the overall aroma. Table S2 of the Supplementary Materials lists the detection thresholds reported in the literature for the main compounds found in the samples.

Two tasting sessions were held at 3 and 6 months after bottling. Both were conducted in a standardized room (UNE-EN-ISO 8589-210) [27] at a temperature of 20 °C. The tasting panel consisted of 14 judges semi-trained for white wine evaluation. Six attributes were selected to be scored: white fruit, stone fruit, tropical fruit, flowery, mint and citrus. The panelists were asked to rate these attributes according to a 0 to 5 scale. Aromatic evaluation was carried out by means of a descriptive tasting (ISO 8587:2006) [28] of the wines obtained from the application of ultrasound compared with the control wine.

### 2.5. Statistical Analyses

The significant differences observed according to the ultrasound application method and time were evaluated by means of a two-way analysis of variance (Two-way ANOVA) together with pairwise tests performed on the data related to aromatic compounds determined by SPE-GC-MS. Similarly, in order to evaluate the data obtained from the sensory analysis, the Kruskal–Wallis nonparametric and Dunn’s post hoc tests were employed to determine whether the wines showed significant differences. In both cases, a 95% confidence interval was used. All the experimental data were analyzed using RStudio software (RStudio, Boston, MA, USA).

## 3. Results and Discussion

### 3.1. Effect on Terpenic Compounds

**Figure 1:**
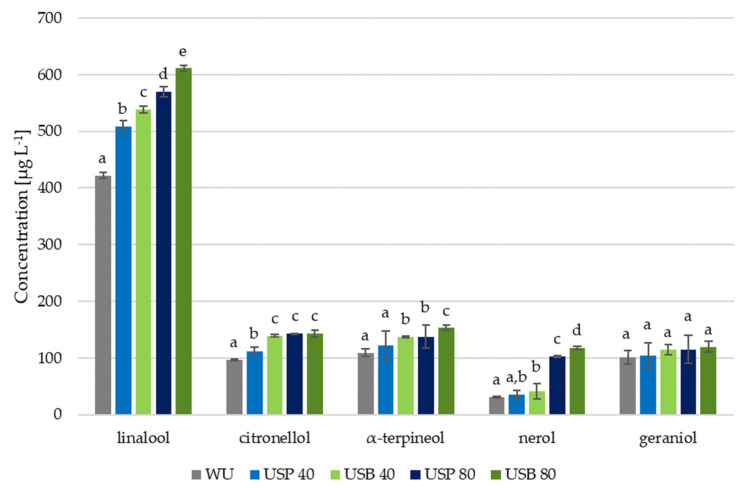
Mean concentrations (*n* = 2) of terpenic compounds in the wines produced with ultrasound application (USB and USP) and in the wines produced without ultrasound application (WU). Bars with different letters indicate significant differences (*p* < 0.05) in the pairwise test results.

Terpenic compounds are the compounds most closely related to the varietal aroma of the Muscatel variety. Figure 1 shows the concentrations of the terpenes that were characterized, as well as the results from the statistical study, which consisted of an ANOVA and a pairwise test.

In this case, regarding most of the terpenic compounds, it can be observed that all the wines that had been elaborated using ultrasound showed significant differences in relation to the wine that had been elaborated without the application of ultrasound (WU). Only geraniol levels presented no significant differences in the different trials. Similarly, it was observed that, with the exception of α-terpineol, the compounds concentration varied in accordance with time and/or ultrasound application, and the interaction between these variables was critical for nerol.

The effect resulting from the application of 40 min of ultrasound was evident both in bath ultrasound (USB) and ultrasonic probe ultrasound (USP) wines. Regarding linalool, the application of 40 min of ultrasound allowed values of 509 ± 1.02 and 538 ± 0.76 µg L^−1^ to be reached, which represents an increment of more than 20% with respect to the wine elaborated without ultrasound (WU). Nerol and α-terpineol also reached increments greater than 20% when bath ultrasound was applied (USB); however, probe ultrasound application did not significantly change α-terpineol levels with respect to the concentrations found in the WU wine.

In the case of 80 min applications during the pre-fermentative maceration, the results were notably more remarkable. For example, citronellol was increased by as much as 48%. Moreover, above all, nerol reached values of 102.67 ± 1.34 µg L^−1^ in USP wines and 117.94 ± 2.91 µg L^−1^ in the USB ones compared with just 31.15 ± 1.62 µg L^−1^ measured in the WU wine.

Therefore, the application of ultrasound either by ultrasound bath or ultrasound probe strongly affects the levels of terpenic compounds. Additionally, the longer the application time, the higher the levels of these compounds. However, the final levels are conditioned by both the type of ultrasound system and the application time.

### 3.2. Ultrasound Effect on Esters

The terpenic compounds are the ones that should present more changes due to the effect of ultrasound during maceration. However, the determination of other relevant compounds that are related to the aromatic characteristics and that could be affected by the application of ultrasound was also addressed. These compounds include ethanolic esters, which are produced after fermentation and that may depend on the levels reached during the maceration by the acids that form them.

**Figure 2:**
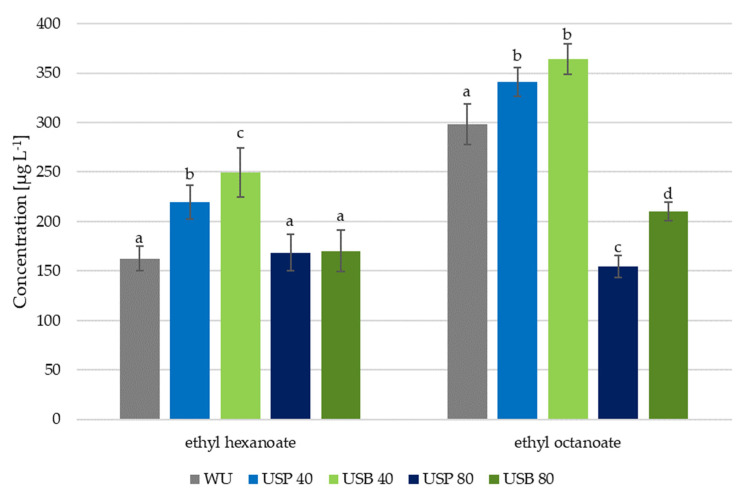
Mean concentrations (*n* = 2) of ester-like compounds in the wines produced using ultrasound application (USB and USP) and in the wines produced without ultrasound application (WU). Different letters over the bars indicate relevant differences.

The concentrations of the two most abundant esters in the samples are shown in Figure 2. As can be seen, with the exception of the concentration of ethyl hexanoate in the wine subjected to 80 min of ultrasound application, all the wines showed significant differences with respect to the wine produced without the application of ultrasound. Likewise, it was observed that the concentration of these compounds depends primarily on ultrasound application time.

The effect from 40 min ultrasound application was clear in both types of ultrasound application methods. In the case of ethyl hexanoate, it went up to 219 ± 17.02 µg L^−1^ in USP wines, while USB wines reached a content as high as 249 ± 20.06 µg L^−1^, which represents respective increments of 27% and 38% compared with the WU wine. However, ethyl octanoate increments remained below 20%.

In the case of 80 min ultrasound application, ester content went down significantly. Thus, USP wines registered 154.48 ± 14.77 µg L^−1^, and USB wines reached just 210.11 ± 9.71 µg L^−1^, which represent a decrease of 48% and 29%, respectively, with respect to the WU wine values at 298.03 ± 11.42 µg L^−1^.

This behavior implies that, although the application of ultrasound during the pre-fermentative maceration diffuses the fatty acids from the grapes’ solid parts into the must, the long periods of application have a certain influence on the stability of the fatty acids, causing them to combine or degrade [21,29,30]. This effect has been observed in red varieties, where the application of 40 and 80 min cycles of ultrasound showed a decrease in fatty acid concentrations in the final wines [31].

### 3.3. Effect of Ultrasound on Hydroxylated Compounds

**Figure 3:**
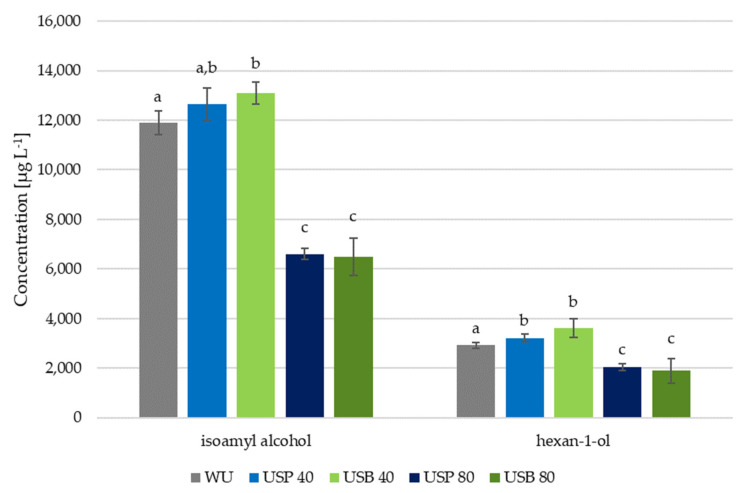
Mean concentrations (*n* = 2) of hydroxylated compounds in wines made using ultrasound (USB and USP) and in wines made without ultrasound (WU). Bars with different letters indicate relevant differences.

Hydroxylated compounds can also be affected by ultrasound maceration. Particularly those related to vegetal aromas. Therefore, the effect of ultrasound application on isoamyl alcohol and on 1-hexanol was evaluated. The former compound comes from the transformation of isoleucine, whose content levels can be affected by maceration, and the latter comes directly from the solid parts in the grapes. The concentrations determined for these two compounds, together with the statistical results, are shown in Figure 3. As can be seen, the application of ultrasound to the wines showed clear differences in all the cases with respect to the wine produced without ultrasound. It was also observed that the wines’ composition depended, to a large extent, on ultrasound application time.

When ultrasound was applied for 40 min, both USB wines presented noticeable differences in their content of both compounds with respect to the WU wine. The increments in hexan-1-ol content reached 24%, while non-significant differences were found for USP wines. Isoamyl alcohol also went up with respect to the WU wine, although just by 10% in USB wines and by 6% in the case of USP wines.

When ultrasound was applied for 80 min, the opposite effect could be observed, with a clear reduction in the content of hydroxylated compounds of both wines treated with ultrasound, regardless of the ultrasound application method. It has been demonstrated that the application of ultrasound to wines can effectively reduce the level of similar compounds, including n-proanol, n-pentanol and the two isomers of isoamyl alcohol [30]. In the resulting wines, the level of isoamyl alcohol registered was 6607.78 ± 232 µg L^−1^ in USP wines, while its content in USB wines went down to 6500.66 ± 758 µg L^−1^. These levels were just around 50% of those registered in WU wines, with values as high as 11,880.48 ± 480 µg L^−1^. Similarly, hexan-1-ol also went down by around 35% to 2052 ± 131 µg L^−1^ in USP wines and to just 1893.35 ± 487 in USB wines against the 2917.23 ± 108 µg L^−1^ content in the WU wine.

### 3.4. Odor Activity Value (OAV)

The OAV, i.e., the concentration of a compound within its threshold of perception, was used to evaluate the aromas. When this value is >1, the compound is perceived individually, whereas if the value is <1, the compound is not individually perceived, but it could be perceived as one of the contributors to the wine aroma as a whole.

According to the data in Table 2 for terpenic compounds, only linalool (OAV > 16) and geraniol (OAV > 3.4) would be individually perceived in all the wines produced. One noteworthy aspect is the case of citronellol, which did not individually influence the aroma of the wine without ultrasound application (OAV < 1) but did influence the aroma of the wines resulting from the application of either probe or bath ultrasound (OAV > 1). This is due to the 40% higher concentration obtained in bath and probe ultrasound wines—except for 40 min USP wines (+15%)—compared with the wine produced without ultrasound. This compound is related to floral [31] and citrus aromas [32]. Therefore, this significant content increment implies that the wines elaborated with ultrasound application may have a differentiated character with respect to those obtained through conventional methods since at least this compound in particular would be incorporated to their aromatic profile.

Regarding the hydroxylated compounds studied, i.e., isoamyl alcohol and 1-hexanol, it was observed that only the latter individually influenced the aromatic profile of the wines. Thus, the wines after 40 min ultrasound application had a higher OAV than that of the WU wines, whereas 80 min ultrasound application wines had a lower OAV than the wine produced without ultrasound.

Finally, of the esters studied, only ethyl hexanoate individually influenced the final wine aroma with an OAV > 32. This compound is related to fruit aromas, especially white and/or tropical fruit [31,33]. As can be seen, in all the cases, it contributed specifically to the aroma, and the application of ultrasound had no effect on the contribution of this compound to the aroma.

Thus, after the analysis of the main volatile compounds related to aroma, the wines produced with the application of ultrasound exhibited higher levels of esters and terpenes, which are responsible for the wines’ fruity and varietal aromas.

### 3.5. Sensory Analysis

Two descriptive tastings of the wines resulting from the application of ultrasound by means of bath or probe and the wine that had not been applied ultrasound were carried out. Table 3 shows the judges’ scores for the different wines.

**Figure 4:**
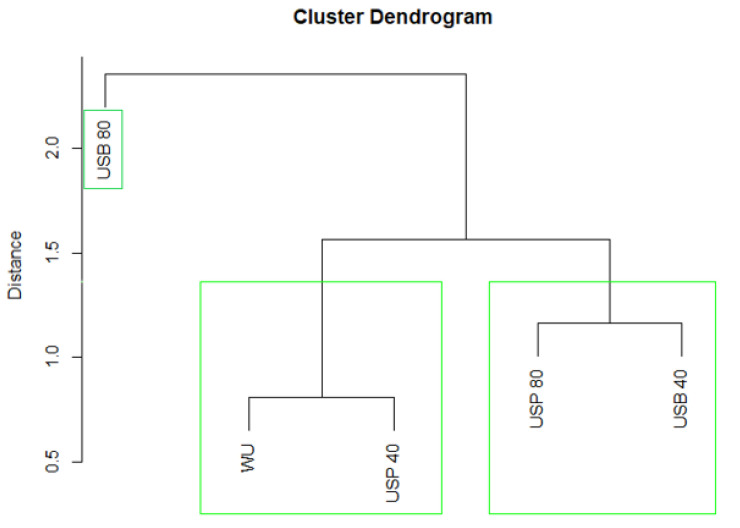
Dendrogram based on the mean scores granted by the judges in the descriptive analysis of the wines.

First, based on the results from the descriptive analysis, a cluster analysis was conducted. Figure 4 shows the resulting cluster analysis diagram, where it can be seen that the wine produced after 80 min bath ultrasound treatment was significantly different from the rest, while the differences were minimal between the wines subjected to 40 min bath ultrasound and 80 min probe ultrasound.

To determine the degree of reliability of the differences reported by the judges, a statistical study on the aromatic descriptors was carried out using the Kruskal–Wallis nonparametric and Dunn’s post hoc tests. Table 4 shows the descriptors that presented significant differences between the wines subjected to ultrasound and the wine without ultrasound. Thus, it was observed that the stone fruit descriptor presented significant differences in all the wines regardless of the ultrasound application time or method. On the other hand, the white fruit and flowery descriptors were only present in the wines treated with 80 min of ultrasound—either bath or probe—and in those treated with 40 min of ultrasound probes. In general, it can be seen that the application of 80 min of ultrasound, either probe or bath, results in greater aromatic differences with respect to the control wine.

**Figure 5:**
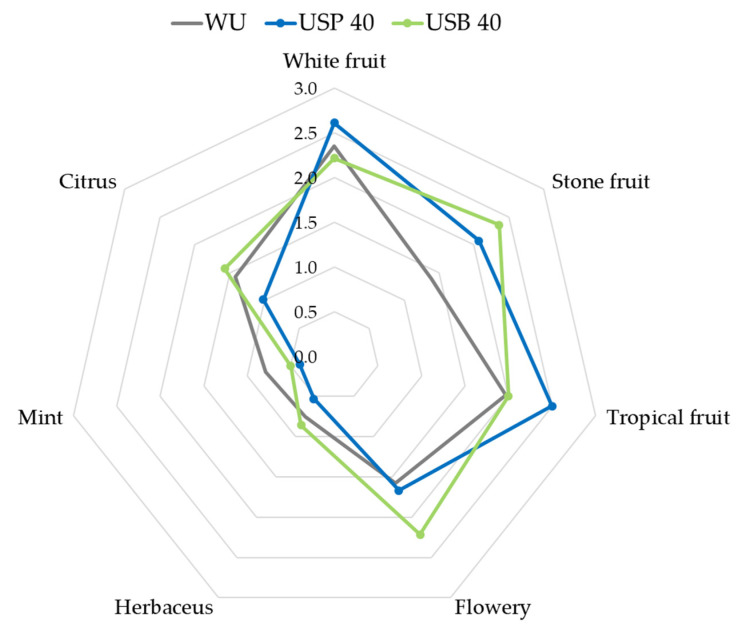
Graphic representation of the organoleptic profiles of the wines resulting from the application of 40 min of ultrasound probe (USP) and ultrasound bath (USB) compared with the wine without ultrasound application (WU).

Figure 5 presents a spider graph based on the results of the wines produced with the application of 40 min ultrasound—probe and bath—vs. the wine produced without ultrasound. As can be seen, the wine resulting from the ultrasound probe application stands out for its aromatic intensity of white fruit, tropical fruit and stone fruit, while the wine resulting from the bath application presents more aromatic intensity of stone fruit and flowers.

**Figure 6:**
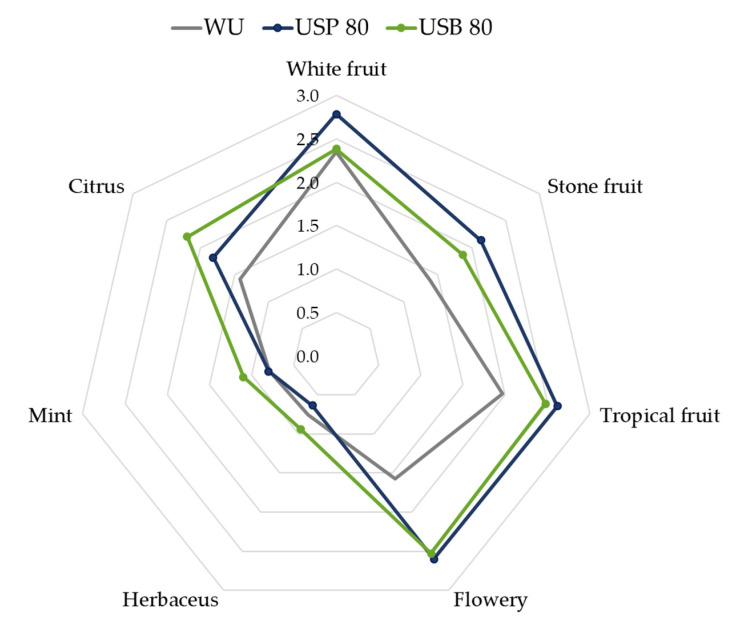
Graphic representation of the organoleptic profiles of the wines resulting from the application of 80 min of ultrasound probe (USP) and ultrasound bath (USB) compared with the wine without ultrasound application (WU).

Similarly, in Figure 6, we can see how the application of 80 min of ultrasound compared with the wine without ultrasound application resulted in different aromatic profiles. For both types of ultrasound application methods, a notable increase in the wines’ flowers, tropical, white and stone fruits aromas was observed. These aromas are related to terpene citronellol which, by means of OAV, was verified to have an individual influence on the wines that had been elaborated using ultrasound.

When comparing Figure 5 and Figure 6, we can conclude that the main effect of the increased maceration time is a greater differentiation between, on the one hand, the two wines elaborated using ultrasound and, on the other hand, the wine elaborated without applying ultrasound. That is, regardless of the ultrasound application method, there is an intensification of the aromatic character as a consequence of the application of ultrasound.

## 4. Conclusions

In general, it can be seen that the application of ultrasound during the pre-fermentative maceration process favors the release and extraction of volatile compounds in greater amounts compared with a conventional pre-fermentative maceration process where no ultrasound is applied. In the particular case that was studied in this research on the Muscat of Alexandria variety, an evident effect from the application of either ultrasound application technique—probe or bath—could be observed. Thus, the wines that were produced using 40 and 80 min of ultrasound were not only distinguishable between themselves, but also from the wine that had been produced without any ultrasound application.

Regardless of the ultrasound application time or method, terpenes in particular—a crucial compound with respect to wine aroma profile—increased substantially with the application of ultrasound, with a notable 200% nerol content increment in the wines subjected to 80 min ultrasound application with respect to the control wine. With regard to the other compounds of interest, which are not directly extracted from the grapes’ solid parts but through derivatives such as ethanolic esters and hydroxylated compounds, an increase was generally registered when 40 min ultrasound was applied, and a general decrease was registered when the application time went up to 80 min.

At an organoleptic level, the most important differences were found in 80 USB wines, which were the most notably differentiated from the rest of the wines elaborated with ultrasound.

For all of the above-mentioned, we can conclude that the application of ultrasound during the maceration process of Muscat of Alexandria musts favors a significantly higher recovery of terpenes from the solid parts of the grapes, which results in a noticeable modification of the aromatic descriptors in the final wines. Consequently, by varying the ultrasound application time, different aromatic profiles can be obtained, given that longer application times intensify some aromatic characteristics and diminish others.

## Figures and Tables

**Table 1:** Equation of the line obtained by means of the calibration line for the different compounds analyzed and their quantification limits (ng L^−1^).

Compound	Equation of Regression	R^2^	LOQ (ng L^−1^)
Linalool	y = 91899.58x + 909.95	0.9999	74
α-terpineol	y = 85289.17x + 843.80	0.9999	73
Nerol	y = 156258.42x + 1236.21	0.9999	45
Geraniol	y = 193469.63x + 760.11	0.9998	79
Citronellol	y = 92791.07x + 441.05	0.9999	140
Isoamyl alcohol	y = 111218.10x − 238.80	0.9983	38
Ethyl hexanoate	y = 121698.30x − 119.89	0.9993	17
Hexan-1-ol	y = 74080.25x − 111.87	0.9990	127
Ethyl octanoate	y = 15487.65x − 337.21	0.9993	98

**Table 2:** Odor Activity Value (OAV) of the different compounds analyzed.

Compound	WU	USP 40	USB 40	USP80	USB 80
Linalool	16.7	20.4	21.5	22.8	24.5
α-terpineol	0.4	0.5	0.6	0.5	0.7
Nerol	0.1	0.1	0.1	0.3	0.3
Geraniol	3.4	3.5	3.8	3.8	4.0
Citronellol	0.9	1.1	1.4	1.5	1.6
Isoamyl alcohol	0.4	0.4	0.4	0.2	0.2
Ethyl hexanoate	2.7	3.0	3.3	1.8	1.7
Hexan-1-ol	32.5	43.9	49.9	33.7	34.1
Ethyl octanoate	0.5	0.6	0.6	0.3	0.4

**Table 3:** Mean scores (*n* = 14) granted by the judges to the ultrasound probe (USP), ultrasound bath (USB) and wines without ultrasound (WU).

Descriptor	WU	USP 40	USB 40	USP80	USB 80
	Mean ± SD	Mean ± SD	Mean ± SD	Mean ± SD	Mean ± SD
White fruit	2.3 ± 1.5	2.6 ± 0.7	2.2 ± 0.8	2.8 ± 0.9	2.4 ± 1.0
Stone fruit	1.4 ± 1.0	2.1 ± 1.5	2.4 ± 1.0	2.1 ± 1.2	1.9 ± 0.9
Tropical fruit	2.0 ± 1.1	2.5 ± 1.0	2.1 ± 1.0	2.6 ± 1.3	2.5 ± 0.7
Flowery	1.6 ± 1.1	1.7 ± 1.2	2.2 ± 1.1	2.6 ± 0.7	2.5 ± 1.2
Mint	0.8 ± 1.1	0.4 ± 0.5	0.5 ± 0.8	0.8 ± 0.9	1.1 ± 1.5
Citrus	1.4 ± 0.8	1.0 ± 1.3	1.6 ± 1.0	1.8 ± 1.4	2.2 ± 0.9

**Table 4:** Results of the Kruskal–Wallis nonparametric and Dunn’s post hoc tests among the wines produced with ultrasound and the wines produced without ultrasound.

Descriptor	USP 40 vs. WU	USB 40 vs. WU	USP 80 vs. WU	USB 80 vs. WU
White fruit				
Stone fruit				
Tropical fruit				
Flowery				
Mint				
Citrus				

Grey: significant differences. White: non-significant differences.

## Data Availability

Data available on request.

## Supplementary Materials

The following are available online at https://www.mdpi.com/article/10.3390/foods10071462/s1, Table S1: Recovery study and standard deviation (SD) of the different compounds analyzed by SPE-GC-MS, Table S2: Odor threshold (µL^−1^) of the different volatile compounds analyzed.
