# Supplementary material for: Changes in the Aromatic Compounds Content in the Muscat Wines as a Result of the Application of Ultrasound during Pre-Fermentative Maceration
Source: Foods. 2021 Jun 24;10(7):1462. doi: 10.3390/foods10071462 (PMC8306965; doi:10.3390/foods10071462)
Supplement: Supplementary file 1 [file foods-10-01462-s001.zip › foods-1262140-SI.pdf]

Table S1. Percentage recovery and standar desviation (SD) for the different compunds analysed by SPE-GC-MS.

Compound	Recovery (%)	SD
Linalool	89	1.32
α-terpineol	93	0.49
Nerol	98	5.12
Geraniol	89	5.10
Citronellol	98	0.78
Isoamyl alcohol	102	8.99
Ethyl hexanoate	129	3.89
Hexan-1-ol	113	5.02
Ethyl octanoate	121	9.30

Table S2 Odor threshold (μg L^{-1}) for the differents volatile compounds analysed.

Compound	Odor threshold	Reference
Linalool	25	[1,2]
α-terpineol	250	[3,4]
Nerol	400	[5]
Geraniol	30	[6,7]
Citronellol	100	[7,8]
Isoamyl alcohol	30,000	[9,10]
Ethyl hexanoate	5	[2,8]
Hexan-1-ol	1100	[1]
Ethyl octanoate	580	[11]

References

1. Castro Vázquez, L.; Pérez-Coello, M.S.; Cabezudo, M.D. Effects of enzyme treatment and skin extraction on varietal volatiles in Spanish wines made from Chardonnay, Muscat, Airén, and Macabeo grapes. *Anal. Chim. Acta* **2002**, *458*, 39–44, doi:10.1016/S0003-2670(01)01521-5.
2. Selli, S. Aroma components of cv . Muscat of Bornova wines and influence of skin contact treatment. *Food Chem.* **2006**, *94*, 319–326, doi:10.1016/j.foodchem.2004.11.019.
3. Zalacain, A.; Marín, J.; Alonso, G.L.; Salinas, M.R. Analysis of wine primary aroma compounds by stir bar sorptive extraction. *Talanta* **2007**, *71*, 1610–1615, doi:10.1016/j.talanta.2006.07.051.
4. Gómez-Míguez, M.J.; Gómez-Míguez, M.; Vicario, I.M.; Heredia, F.J. Assessment of colour and aroma in white wines vinifications: Effects of grape maturity and soil type. *J. Food Eng.* **2007**, *79*, 758–764, doi:10.1016/j.jfoodeng.2006.02.038.
5. González Álvarez, M.; González-Barreiro, C.; Cancho-Grande, B.; Simal-Gándara, J. Relationships between Godello white wine sensory properties and its aromatic fingerprinting obtained by GC-MS. *Food Chem.* **2011**, *129*, 890–898, doi:10.1016/j.foodchem.2011.05.040.
6. Palomo, E.S.; Díaz-maroto, M.C.; Viñas, M.A.G.; Soriano-pérez, A.; Pérez-coello, M.S. Aroma profile of wines from Albillo and Muscat grape varieties at different stages of ripening. *Food Control* **2007**, *18*, 398–403, doi:10.1016/j.foodcont.2005.11.006.
7. Caro, del; Fanara, C.; Genovese, A.; Moio, L.; Piga, A.; Piombino, P. Free and enzymatically hydrolysed volatile compounds of sweet wines from Malvasia and Muscat grapes (Vitis vinifera L.) grown in Sardinia. *South African J. Enol. Vitic.* **2012**, *33*, 115–121, doi:10.21548/33-1-1313.

8. Mayén, M.; Zea, L.; Mérida, J.; Moyano, L.; Toledano, A.; Medina, M. Compuestos fenólicos y del aroma diferenciadores de vinos generosos tipo fino y oloroso - Dialnet. *Rev. Enólogos*, **2005**, *36*, 30–33.
9. Sánchez-gómez, R.; Zalacain, A.; Pardo, F.; Alonso, G.L.; Salinas, M.R. Moscatel vine-shoot extracts as a grapevine biostimulant to enhance wine quality. *Food Res. Int.* **2017**, *98*, 40–49, doi:10.1016/j.foodres.2017.01.004.
10. Guth, H. Quantitation and Sensory Studies of Character Impact Odorants of Different White Wine Varieties. *J. Agric. Food Chem.* **1997**, *45*, 3027–3032, doi:10.1021/jf970280a.
11. Peinado, R.A.; Moreno, J.; Bueno, J.E.; Moreno, J.A.; Mauricio, J.C. Comparative study of aromatic compounds in two young white wines subjected to pre-fermentative cryomaceration. *Food Chem.* **2004**, *84*, 585–590, doi:10.1016/S0308-8146(03)00282-6.
